# Body mass index and incidence of nonaggressive and aggressive prostate cancer: a dose-response meta-analysis of cohort studies

**DOI:** 10.18632/oncotarget.20930

**Published:** 2017-09-15

**Authors:** Bo Xie, Guanjun Zhang, Xiao Wang, Xin Xu

**Affiliations:** ^1^ Department of Urology, Tongde Hospital of Zhejiang Province, Hangzhou, Zhejiang 310012, China; ^2^ Department of Urology, Hospital of Traditional Chinese Medicine of Shangyu, Shangyu 312300, Zhejiang, China; ^3^ Department of Urology, First Affiliated Hospital, School of Medicine, Zhejiang University, Hangzhou, 310003, China

**Keywords:** body mass index, prostate cancer, meta-analysis, dose-response, cohort

## Abstract

The relationship between body mass index (BMI) and incidence of prostate cancer is still inconclusive. We performed a dose-response meta-analysis of eligible cohort studies to evaluate potential association of BMI with prostate cancer risk by subtype of prostate cancer (nonaggressive and aggressive). A comprehensive literature search was performed in PubMed and Web of Science databases through March 22, 2017. Linear and non-linear dose-response meta-analyses were carried out to evaluate the effects of BMI on incidence of prostate cancer. A total of 21 cohort or nested case-control studies (17 for nonaggressive and 21 for aggressive prostate cancer) were included in this meta-analysis. For nonaggressive prostate cancer, the pooled relative risk (RR) per 5 kg/m^2^ increment of BMI with 95% confidence interval (CI) was 0.96 (95% CI 0.92–1.00). Sensitivity analysis indicated that this result was not robust and steady. For aggressive prostate cancer, a significant linear direct relationship with BMI (RR, 1.07; 95% CI 1.03–1.12) for every 5 kg/m2 increase was observed. Statistically significant heterogeneity was detected for nonaggressive prostate cancer (*P* = 0.020, *I*^*2*^ = 46.1%) but not for aggressive prostate cancer (*P* = 0.174, *I*^*2*^ = 22.4%). In conclusion, BMI level may be positively associated with aggressive prostate cancer risk. Further large prospective cohort studies are warranted to confirm the findings from our study.

## INTRODUCTION

Prostate cancer has become the most common malignancy in males in several developed countries and the second most common one worldwide after lung cancer [1]. The precise etiology of prostate cancer is still virtually unknown and the only well-established risk factors are those that are inherited and uncontrollable, including age, race, and family history of prostate cancer [2]. Emerging evidence indicates that environmental factors may also play an important role in the carcinogenesis and progression of prostate cancer. A high incidence of prostate cancer in the USA and European countries suggests that prostate cancer may be related to the ‘‘Western’’ lifestyle pattern [3].

To date, a large number of well-designed prospective cohort studies have been performed to evaluate the potential relationship between body mass index (BMI) and prostate cancer risk with positive, negative, or null results. Several studies indicated that body adiposity may have a dual effect on two subtypes of prostate cancer (localized/non-aggressive and advanced/aggressive cancer) [4, 5]. Therefore, a recent meta-analysis published in 2012 reviewed all eligible cohort studies investigating this topic and assessed the potential association separately by tumor characteristics. As a result, a high BMI appeared to increase the risk of advanced prostate cancer while reducing the risk of localized disease. Thus, these results support the hypothesis of a dual effect of BMI on prostate cancer carcinogenesis [6]. Since then, more high-quality cohort studies [7–12] have been performed on this topic separately by tumor grade. However, the results are still controversial.

In this study, we performed an updated random-effects dose-response meta-analysis of all available cohort studies up to now in order to comprehensively evaluate the association between BMI and incidence of prostate cancer separately by tumor characteristics.

## RESULTS

### Literature search and study characteristics

**Figure 1:**
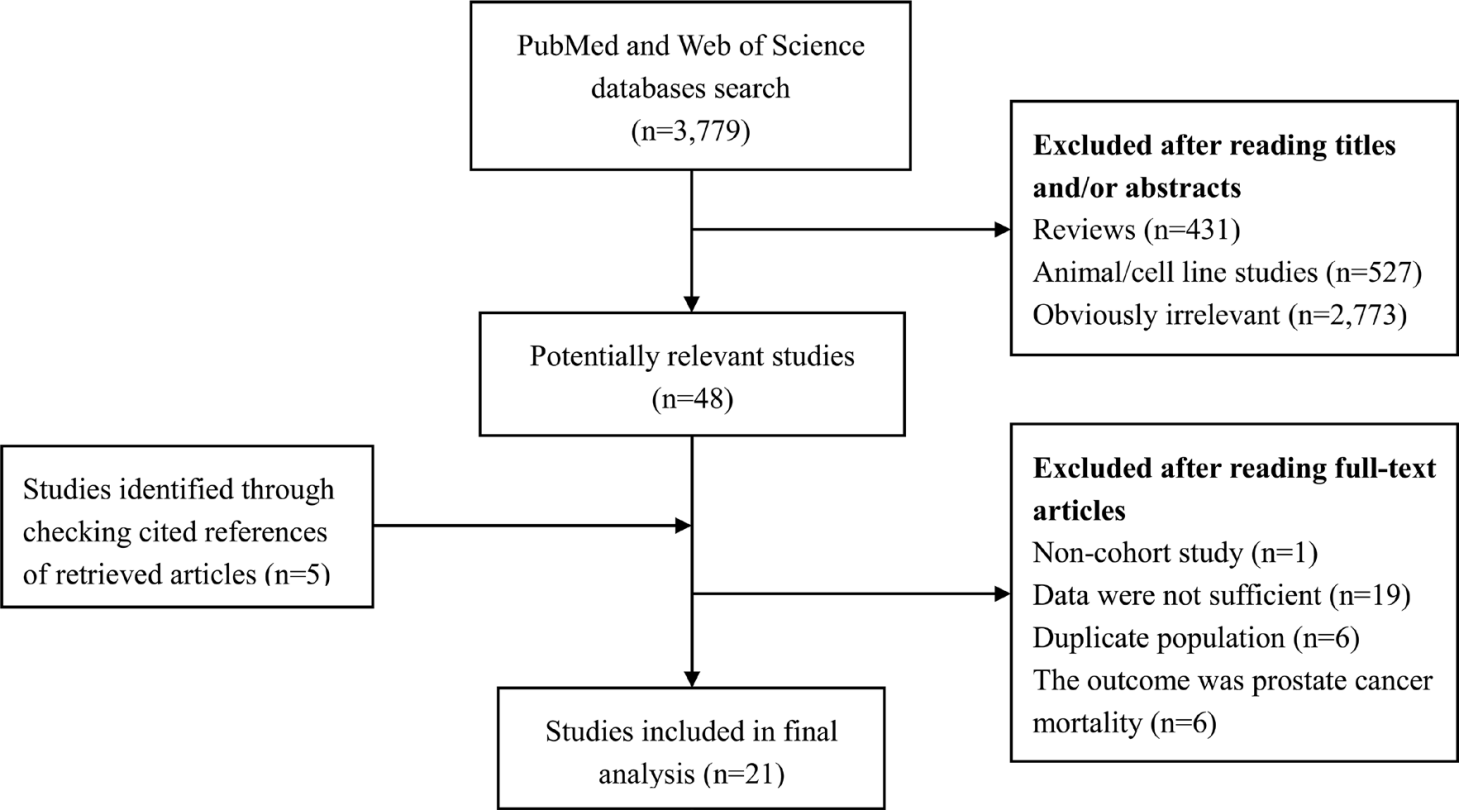
Literature search and study selection PubMed and Web of Science databases were searched from their inception to March 22, 2017. 21 eligible studies were finally included in the present meta-analysis.

The detailed process of literature search is shown in Figure 1. 21 eligible studies [4, 5, 7–25] were finally included in the present meta-analysis. These studies were published between 1994 and 2017 with cohort size ranging from 1,050 to 336,159. The studies were completed in the following geographical regions: Europe (*n =* 8), North America (*n =* 11), Asia (*n =* 1), and Oceania (*n =* 1). Study quality assessment yielded an average score of 6.67. Additional information on the included studies is available in Table 1.

**Table 1:** Main characteristics of studies included in this meta-analysis

Author, year	Country	No. of cases	No. of cohort	Age	Study name or source	Duration of follow-up	Quality score	Adjustment factors
Kelly et al., 2017 [7]	USA	7,822	69,873	62 (55-74)	PLCO Cancer Screening Trial	13 y	7	Age, trial arm, screening center, race, education, married or cohabiting, diabetes, smoking, PSA history, family history of prostate cancer, and myocardial infarction
Bonn et al., 2016 [8]	Sweden	735	15,827	65.2 (SD 10.1)	STHLM-2 cohort	3.5 y	6	Age, physical activity, education, smoking, stress, family history of prostate cancer
Grotta et al., 2015 [10]	Sweden	904	13,109	55.1 (SD 15.2)	Swedish National March Cohort	13 y	7	Age, physical activity, education, smoking, alcohol, and diabetes
Møller et al., 2015 [9]	Denmark	1,813	26,877	50-64	Diet, Cancer and Health Study	15.5 y	7	Age
Rundle et al., 2013 [11]	USA	494	6,692	65.85	Henry Ford Health System	1990–2007	7	Age, race, follow-up duration, biopsy versus TURP, date of procedure, PSA levels, family history of PCa, the number of PSA tests and DRE
Bassett et al., 2012 [12]	Australia	1,374	16,514	68 (47-86)	Melbourne Collaborative Cohort Study	15 y	8	Age, country of birth, and education
Discacciati et al., 2011 [14]	Sweden	2,084	36,959	45-79	Central Sweden	1998–2008	7	Age, energy intake, physical activity, education, smoking, family history of PCa, personal history of diabetes, and BMI at age 30 years
Stocks et al., 2010 [23]	Sweden	10,002	336,159	34.7 ± 13.1	Swedish Construction Workers cohort	22.2 y	6	Age, birth year, smoking, and blood pressure
Wallström et al., 2009 [24]	Sweden	817	10,564	45-73	Malmo Diet and Cancer Study	11 y	7	Age, height, co-habitation status, socioeconomic status, alcohol, smoking, prevalent diabetes, physical activity, birth country, and total intake of EPA, DHA, red meat, and calcium
Pischon et al., 2008 [20]	Eight European countries	2,446	129,502	52.8 (25-70)	European Prospective Investigation into Cancer and Nutrition	8.5 y	8	Age, study center, education, smoking, alcohol, physical activity, and height
Littman et al., 2007 [19]	USA	832	34,754	50-76	Vitamins and Lifestyle Study	2000–2004	6	Age, family history of PCa, race, and PSA screening in the 2 years before baseline
Rodriguez et al., 2007 [4]	USA	5,252	69,991	50-74	Cancer Prevention Study II	1992–2003	8	Age, race, education, family history of PCa, total calorie intake, smoking, history of PSA testing, history of diabetes, and physical activity
Wright et al., 2007 [25]	USA	9,986	287,760	50–71	NIH-AARP Diet and Health Study	5 y	6	Age, race, smoking, education, personal history of diabetes, and family history of PCa
Gong et al., 2006 [5]	USA	1,936	10,258	≥ 55	Prostate Cancer Prevention Trial	7 y	7	Age, race, treatment, diabetes, and family history of PCa
Kurahashi et al., 2006 [17]	Japan	311	49,850	40-69	Japan Public Health Centre-based Prospective Study	1990–2003	7	Age, area, smoking, family history of PCa, and marital status
Habel et al., 2000 [16]	USA	2,079	70,712	18-84	Kaiser Permanente Medical Care Program	19.5 y	7	Age, race, and birth year
Putnam et al., 2000 [21]	USA	101	1,572	68.1 (40-86)	A Cohort of Iowa Men	1986–1995	6	Age, total energy, carbohydrates, linoleic acid, lycopene, retinol, red meat, and family history of PCa
Schuurman et al., 2000 [22]	Netherland	681	58,279	55-69	Netherlands Cohort Study	6.3 y	6	Age, family history of PCa, and socioeconomic status
Cerhan et al., 1997 [13]	USA	71	1,050	65-101	Iowa 65+ Rural Health Study	1982–1993	5	Age, smoking, and physical activity
Giovannucci et al., 1997 [15]	USA	1,369	47,781	40-75	Health Professionals Follow-Up Study	1986–1994	5	Age, height, and BMI at age 21
Le Marchand et al., 1994 [18]	USA	198	20,316	≥ 18	Hawaii	1975–1989	7	Age, ethnicity, and income

BMI, body mass index; No., number; y, years; PCa, prostate cancer; NA, not available; PLCO, Prostate, Lung, Colorectal and Ovarian.

### Linear dose-response analysis

**Figure 2:**
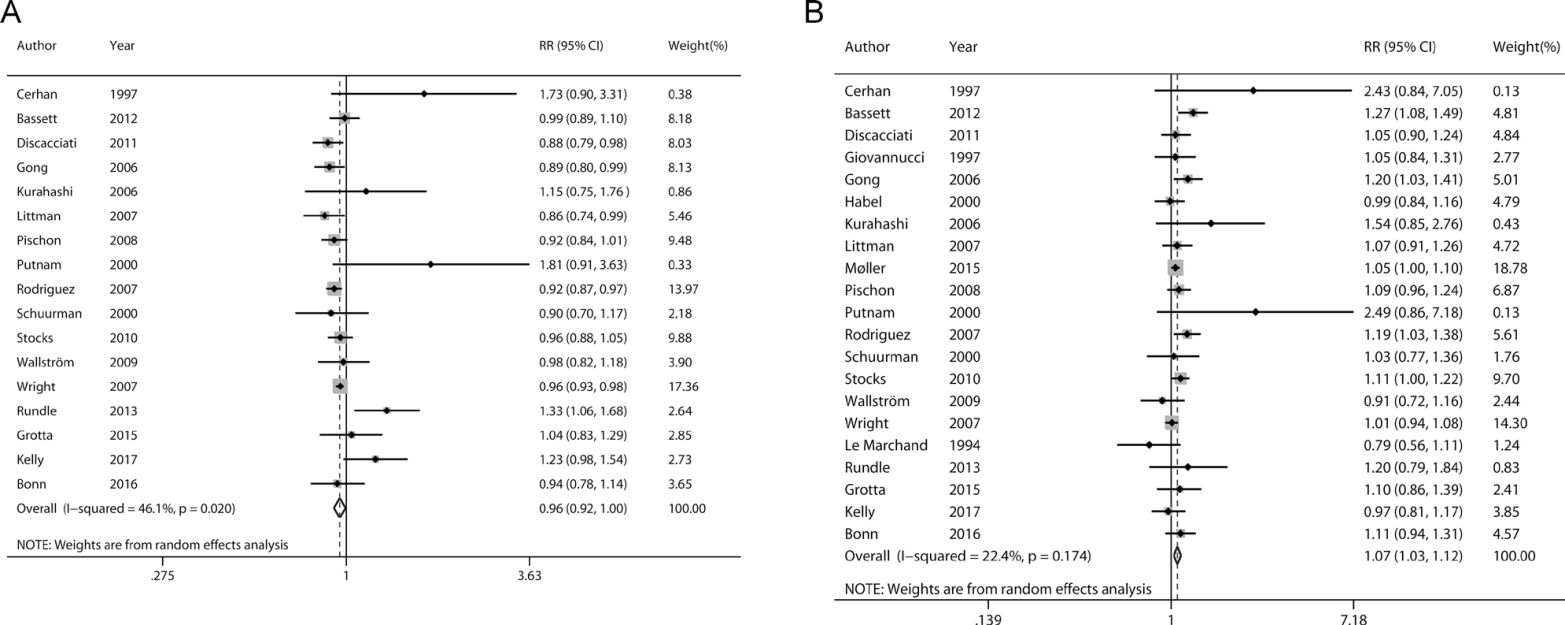
Forrest plots showing RRs of nonaggressive (**A**) and aggressive prostate cancer (**B**) associated with each 5 kg/m^2^ increase in body mass index. The size of each square is proportional to the study’s weight (inverse of variance). Weights are from random effects analysis. Abbreviations: RR, relative risk; CI, confidence interval.

Figure 2 presents the pooled linear dose-response relationship between BMI and incidence of nonaggressive/aggressive prostate cancer. The pooled RRs for 5 kg/m^2^ increment of BMI were 0.96 (95% CI 0.92–1.00) and 1.07 (95% CI 1.03–1.12) for nonaggressive and aggressive prostate cancer, respectively. Statistically significant heterogeneity was detected for nonaggressive prostate cancer (*P* = 0.020, *I*^*2*^ = 46.1%) but not for aggressive prostate cancer (*P* = 0.174, *I*^*2*^ = 22.4%).

### Galbraith plot analysis

**Figure 3:**
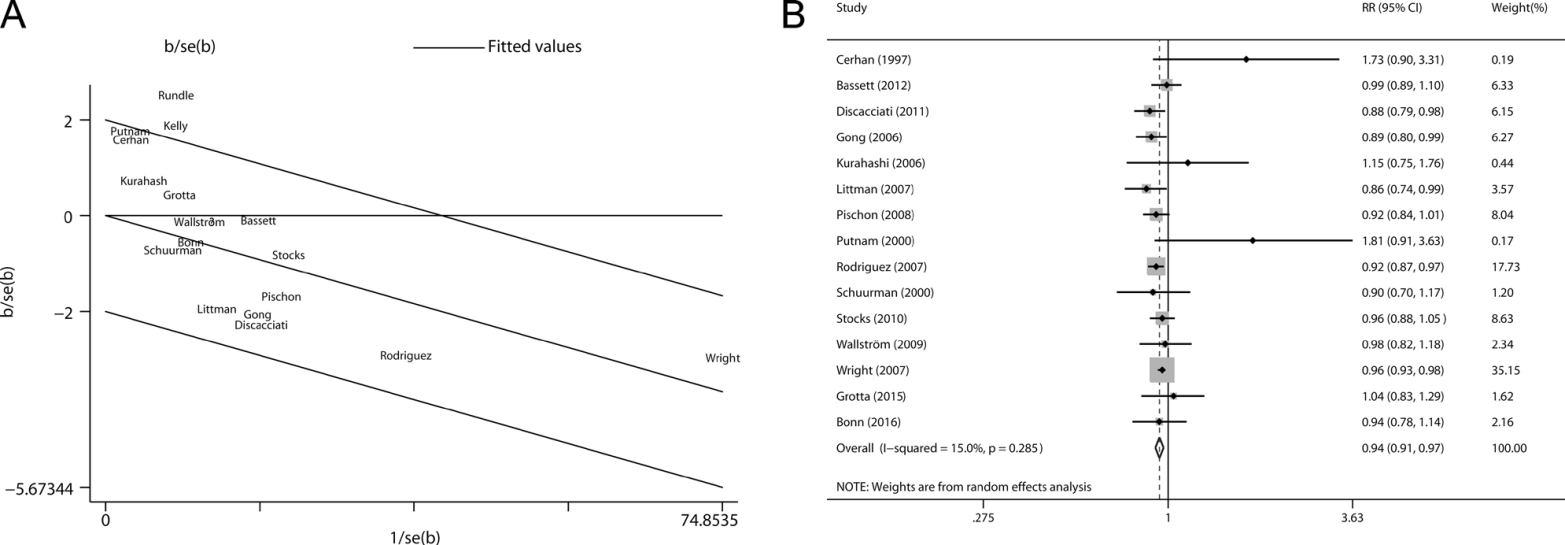
Evaluation of heterogeneity (**A**) Galbraith plot was introduced to explore the potential sources of heterogeneity. As a result, two studies led to the heterogeneity. (**B**) Pooled risk estimate with its 95% CI for the association between BMI and non-aggressive prostate cancer risk after removing the studies that led to heterogeneity.

Galbraith plot was used to detect the studies that contributed to the heterogeneity. As a result, two studies by Kelly et al. and Rundle et al. [7, 11] led to the heterogeneity among those contributing for nonaggressive prostate cancer (Figure 3A). After removing these two studies, the heterogeneity became small (*P* = 0.285, *I*^*2*^ = 15.0%) and the direction of the combined RR did not change (RR = 0.94, 95% CI 0.91–0.97) (Figure 3B).

### Sensitivity analysis

**Figure 4:**
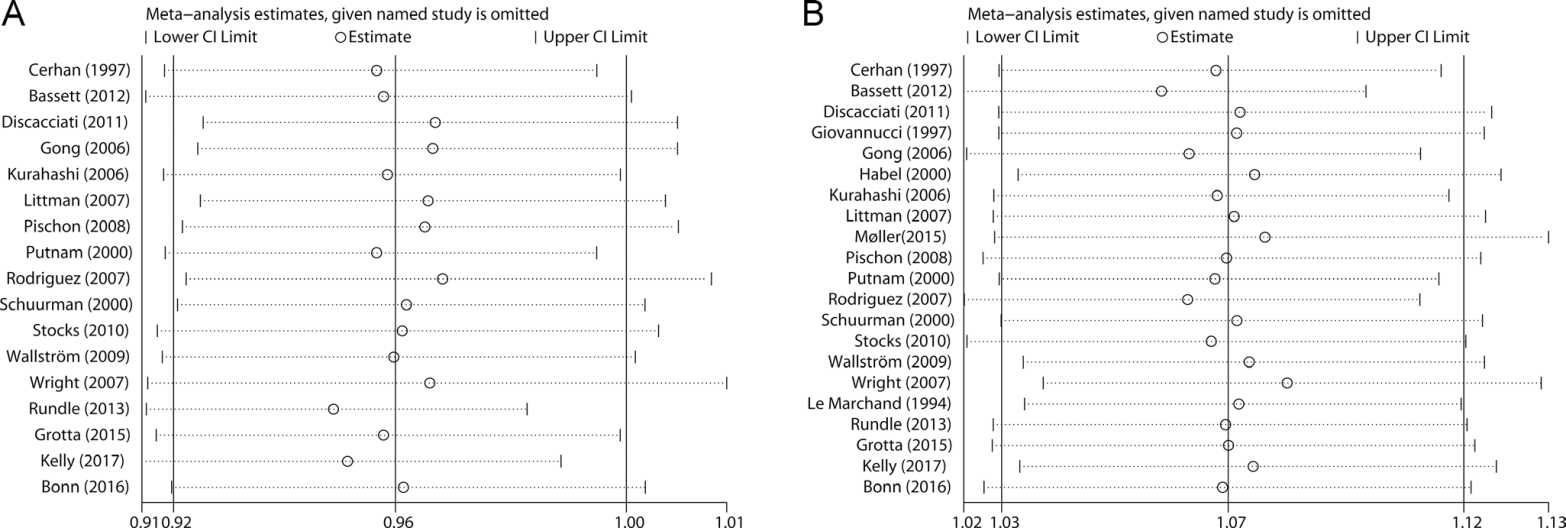
Sensitivity analyses were performed whereby each study was omitted in turn and the pooled risk estimates were recalculated to determine the influence of each study (**A**) nonaggressive prostate cancer; (**B**) aggressive prostate cancer.

In the sensitivity analysis, we removed each included study at a time and repeated the meta-analysis. The combined RRs for non-aggressive prostate cancer were not robust and steady (Figure 4A). In contrast, the pooled risk estimates for aggressive prostate cancer were not influenced greatly by any individual study (Figure 4B).

### Publication bias

**Figure 5:**
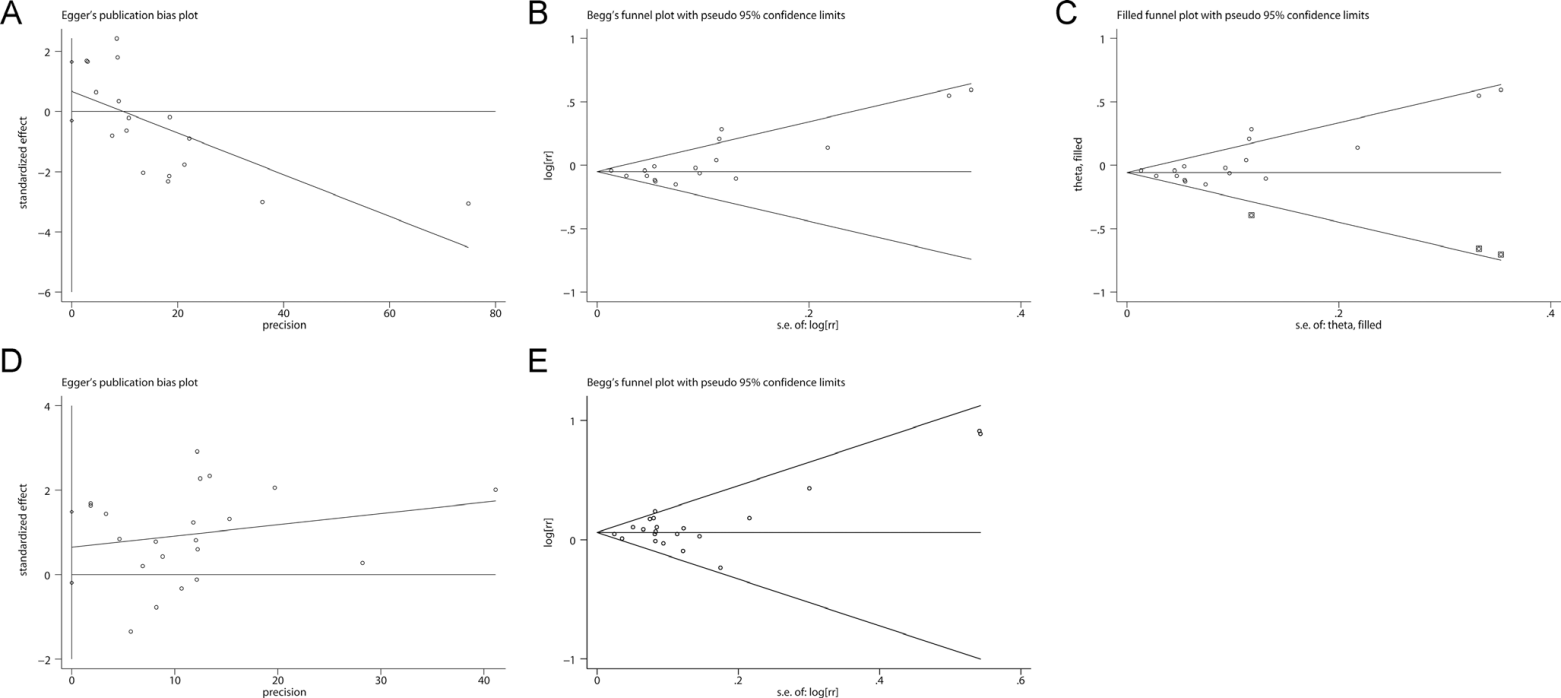
Evaluation of publication bias Potential publication bias was detected for non-aggressive prostate cancer. No significant evidence of publication bias was observed for aggressive prostate cancer. (**A**) Begg’s test for non-aggressive prostate cancer; (**B**) Egger’s test for non-aggressive prostate cancer; (**C**) Trim-and-fill analysis for non-aggressive prostate cancer; (**D**) Begg’s test for aggressive prostate cancer; (**E**) Egger’s test for aggressive prostate cancer.

Potential publication bias was detected for non-aggressive prostate cancer (Figure 5A and 5B, Egger’s test*: P* = 0.003; Begg’s test: *P* = 0.044). The application of trim and fill analysis did not virtually change the pooled risk estimate for non-aggressive prostate cancer (Figure 5C, RR = 0.95, 95% CI 0.90–0.99). No significant evidence of publication bias was observed for aggressive prostate cancer in Egger’s test (Figure 5D, *P* = 0.339) or Begg’s test (Figure 5E, *P* = 0.608).

### Nonlinear dose-response analysis

**Figure 6:**
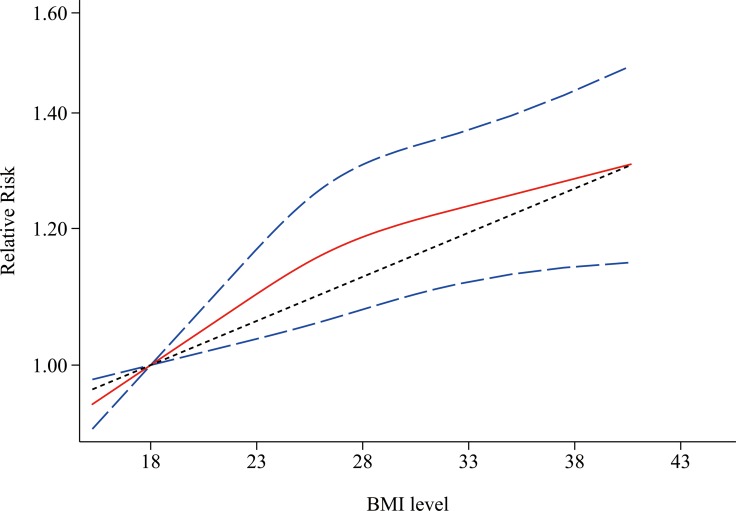
Non-linear dose-response associations between body mass index and relative risk for aggressive prostate cancer Red solid line and blue dash lines represent point estimates and 95% confidence intervals for non-linear analysis; Grey dash line represents point estimates for linear analysis. Abbreviations: BMI, body mass index.

Figure 6 shows the results of non-linear dose-response meta-analysis for aggressive prostate cancer. There was no evidence of a nonlinear relationship between BMI level and aggressive prostate cancer (*P* = 0.181 for nonlinearity).

## DISCUSSION

This updated dose-response meta-analysis summarized all available cohort studies that explored the relationship between BMI level and incidence of prostate cancer separately by tumor characteristics. As a result, high BMI may be related with an increased risk of aggressive prostate cancer. On the other hand, a borderline inverse relationship between high BMI and nonaggressive prostate cancer was observed, but sensitivity analysis indicated that this result was not robust and steady.

A previous meta-analysis also examined two prostate cancer subtypes (localized and advanced) separately based on 12 and 13 cohort studies, respectively. They observed an inverse linear relationship for localized prostate cancer (RR = 0.94, 95% CI 0.91–0.97 for every 5 kg/m^2^ increase) but a linear positive relationship for advanced prostate cancer (RR = 1.09, 95% CI 1.02–1.16 for every 5 kg/m^2^ increase) [6]. In contrast, although we also found a linear dose-response relationship for aggressive prostate cancer, the evidence that supported the inverse relationship between BMI and nonaggressive prostate cancer was weak.

Several potential mechanisms might mediate the positive association between BMI and risk of aggressive prostate cancer. Obesity leads to high circulating concentrations of insulin, leptin and insulin-like growth factor-I (IGF-I) and low levels of adiponectin, which have been described to promote prostate cancer growth and progression, thus increasing the risk of advanced prostate cancer [26]. In addition, obese men might have a lower concentration of serum testosterone [27]. Although testosterone could stimulate the growth and development of prostate cancer, testosterone could also help maintain the normal differentiated state of the prostate [28]. Hence reduced testosterone may be associated with a higher risk of less differentiated and more aggressive prostate cancer [29].

This meta-analysis has several limitations that should be acknowledged. Firstly, exposure assessment was based on information collected at the baseline. Participants may have changed their BMI over the long follow-up period, which may have led to some bias in risk estimation. Secondly, a meta-analysis is not able to avoid the problems of confounders that could be inherent in the included studies, which may result in either an overestimation or an underestimation of an effect measure. Thirdly, the criteria used to definite nonaggressive and aggressive prostate cancer varied between different cohorts and involved Gleason score, World Health Organization grading system, TNM (tumor-node-metastasis), and so on, which may lead to a misclassification bias. Fourthly, obvious heterogeneity was observed for nonaggressive prostate cancer, which would throw some doubts on the reliability of the pooled estimates. Fifthly, as lack of individual participant data, we are not able to determine the independent effect of individual variables on study outcomes. Sixthly, like all meta-analyses, this study also has the limitation of being a retrospective analysis. Lastly, potential publication bias was detected by Begg’s and Egger’s tests, which may be due to the fact that small studies with null findings tend not to be published.

This present meta-analysis also has some strengths. Linear and non-linear dose-response analyses were adopted to explore the potential relationship between BMI level and prostate cancer risk. Sensitivity analyses and Galbraith plot analysis were performed to assess the robustness and stability of the pooled risk estimates. All included studies were cohort studies and came from several countries. The risk estimates were extracted from the models adjusting for most established risk factors in each study.

In conclusion, this systematic review and dose-response meta-analysis indicates that BMI level is associated with aggressive prostate cancer risk. Further large prospective cohort studies are warranted to confirm the findings from our study.

## MATERIALS AND METHODS

### Literature search

Two investigators (BX and XX) independently searched PubMed and Web of Science databases from their inception to March 22, 2017 with the following keywords: (“body mass index” OR “BMI” OR “body size” OR “overweight” OR “obesity” OR “adiposity”) AND (“prostate cancer” OR “prostate neoplasm”). No language or date restrictions were applied. The reference lists of retrieved studies and related reviews were also checked for additional eligible articles.

### Selection criteria

Studies included in this meta-analysis had to meet all of the following criteria: *i*) cohort or nested case-control studies; *ii*) the exposure was BMI; *iii*) the outcome of interest was incidence rate of prostate cancer; *iv*) risk estimates with 95% CIs were provided.

### Data extraction and quality assessment

Data were extracted independently with a standardized data collection form by two reviewers (BX and XX). The following information were recorded: first author’s surname, publication date, country, cohort name, sample size, the number of cases, follow-up duration, adjusted variables, BMI exposure levels, and corresponding risk estimates with 95% CIs. Quality assessment of each included study was performed with the Newcastle-Ottawa scale (NOS) (http://www.ohri.ca/programs/clinical_epidemiology/oxford.asp). Any discrepancies were resolved by discussing with a third reviewer.

### Statistical methods

RR with its corresponding 95% CI was used to evaluate the relationship between the BMI and incidence of prostate cancer. A random-effects model was adopted to account for both within- and between-study heterogeneity. For dose-response meta-analysis, we assigned the reported median or mean BMI level of each category to the corresponding RR. If medians or means were not available, the midpoint of the lower and upper bounds of that category was adopted. When the highest category was open-ended, the width of the category was assumed to be the same as the closest adjacent category [6]. The method proposed by Greenland and Longnecker [30] was used to estimate the dose-response trend for each study. These dose-response trends were then pooled with a random-effects meta-analysis. Finally, a potential non-linear dose-response relationship was also explored by using restricted cubic regression splines with three knots at the 25th, 50th, and 75th percentiles of the distribution [31].

Publication bias was evaluated by Begg’s test [32], Egger’s test [33] and a trim-fill analysis. Heterogeneity was assessed by Cochran’s Q (significance level was set to *P* < 0.10) and *I*^*2*^ [34]. Galbraith plot was used to identify the studies that contributed to the heterogeneity. Sensitivity analyses were performed by removing one study at a time and recalculating the pooled risk estimate for the remaining studies. Statistical analyses were completed with Stata version 10 (StataCorp, College Station, TX).
